# Promising Clinical Outcome With Long Term Follow-Up After Body Gamma Knife Stereotactic Radiosurgery for Patients With Early Stage Non-small Cell Lung Cancer

**DOI:** 10.3389/fonc.2018.00618

**Published:** 2018-12-21

**Authors:** Hongqi Li, Jing Li, Xuan Wang, Haifeng Pang, Yupeng Di, Gang Ren, Ping Li, Chen Liu, Xiao Chen, Xiaoli Kang, Yingjie Wang, Tingyi Xia

**Affiliations:** ^1^Department of Radiation Oncology, Airforce General Hospital PLA, Beijing, China; ^2^Medical School, People's Liberation Army General Hospital, Beijing, China

**Keywords:** early stage lung cancer, non-small cell lung cancer, body gamma knife, stereotactic body radiotherapy (SBRT), stereotactic ablative radiation therapy (SABR), stereotactic radiosurgery (SRS)

## Abstract

**Introduction:** Stereotactic ablative radiosurgery (SRS) or stereotactic ablative body radiotherapy (SABR) is the standard treatment for patients with inoperable early stage non-small cell lung cancer (NSCLC), the body gamma knife SRS (ɤ-SRS) is a special SABR technology developed in China. This study prospectively assessed the clinical outcome, toxicity and cost following body ɤ-SRS for early stage NSCLC.

**Methods:** From 2007 to 2010, a total of 29 patients with early stage NSCLC were prospectively enrolled in this study. The prescription dose for Planning Target Volume (PTV), Clinical Target Volume (CTV), and Gross Target Volume (GTV) were 50, 60, and 70 gray (Gy) in 10 fractions. Isodose curves of 50, 60, and 70% covered at least 100% of PTV, 90% of CTV, and 80% of GTV, respectively. The body ɤ-SRS was delivered 5 days per week and completed in 2 weeks.

**Results:** Median follow-up time was 62.0 (range 11.1-140.3) months. 1-, 3-, 5-year OS rates were 93.1%, 72.0%, 60.3%; PFS rates were 86.2, 64.2 and 48.8%; and LR, RR, and DM rates were 10.9%, 21.4%, 29.0%. The median cost of the body ɤ-SRS during treatment was 4,838 (range 4,615–4,923) dollars and the median cost through 5 years was 36,960 (range 9920-56,824) dollars.

**Conclusion:** With existing clinical data, the body ɤ-SRS is an effective treatment option for patients with medically inoperable early stage NSCLC or patients who do not prefer operation, as they may benefit from the minimized toxicity. Due to excellent cost effectiveness, the availability of the body ɤ-SRS will expand, especially in developing nations, and underdeveloped countries.

## Introduction

Surgery is the standard of care for patients with medically operable early stage NSCLC, but SRS and SABR have been regarded as the standard treatment for patients with inoperable early stage NSCLC (1–4), even an option for operable patients (5, 6). The first prospective study with 7 years of follow-up investigated the use of SABR for patients with stage I NSCLC. The results indicated promising local control (LC) and low toxicity and, when counting death as a competing risk, the estimated 7-year incidences of local recurrence (LR), regional recurrence (RR), and distant metastases (DM) rates were 8.1, 13.6, and 13.8%, respectively, (7). No patients developed grade 4 or 5 adverse events. Currently, to the best of our knowledge, the cost of SABR is relatively expensive and its application is limited in developing and underdeveloped countries. Thanks to the development of various medical detection technologies, more and more patients are detected in early stage. Due to the effectiveness and safety of SRS and SABR, they were approved and are gradually being applied to patients with inoperable or borderline operable (8) NSCLC as well as to some operable patients who refuse surgery.

The body ɤ-SRS is a new kind of SABR technology developed in China in 1999. It has a high dose rate, excellent focus performance, high energy utilization rate and shorter irradiation time features. It has 30 Co60 radiation sources with a total activity of 8500Ci in a conical surface. Each source has 3 separate collimators with different diameters (1, 3, 5cm) (9). The head of the radiation source is an iron ball rind with 30 Co60 sources scattered throughout the cavity of the primary collimator. During treatment, the body of the radiation source can rotate horizontally around the central axis with the 30 bundles of gamma ray directed toward a focal target. As the aperture diameter of the collimator decreases, the density of the distributed dose increases but the periphery dose decreases. Three groups of terminal collimators with different apertures can direct the focusing. Isodose distribution is densely distributed as concentric circles with a small range of 50% isodose lines (10, 11). Target volumes of 1–3 cm in diameter are the best indications. When multiple foci are used to treat large tumors, by adjusting the aperture diameter of the collimator and the number of foci (each focus covers only a portion of the target volume), it can generate a conformal isodose distribution similar to that of radioactive seed implantation with delivery of the highest dose to the GTV and delivery of a minimized dose to surrounding normal tissues, even a target of 1–10 cm in diameter could be treated (but we do not suggest this for lesions where the diameter is over 5 cm). Xia et al. (9) first reported the 1- and 3-year overall survival (OS) rates were 100 and 91.0% for patients with medically inoperable NSCLC following ɤ-SRS. But the reports on its long-term outcome and cost are missing. Thus, long-term data regarding the outcomes and cost of body ɤ-SRS are needed urgently to strengthen confidence in its use.

We initiated a prospective clinical study of body ɤ-SRS in 2007 (10). The objectives of this study were to assess the therapeutic outcome, toxicity and cost of body ɤ-SRS for patients with medically inoperable early-stage NSCLC or patients who prefer not having an operation.

## Materials and Methods

### Patients and Study Design

From January 2007 to July 2010, a total of 29 patients were prospectively enrolled in this study. Inclusion criteria were a diagnosis of medically inoperable or refused surgery clinical stage I/II histologically confirmed NSCLC and, for patients without pathological or cytological evidence, MDT (multidisciplinary tumor board) diagnosis was mandatory; ECOG performance ≤ 2; patients could keep in supine or prone position for more than 30 min; and all patients were clinically staged by 18F-FDG PET/CT and CT within 1 month before body ɤ-SRS, according to the International Union against Cancer TNM classification system (UICC 2002). The MDT included radiation oncologists, medical oncologists, surgeons, a pathologist, and a radiologist. Patients who previously received chemotherapy or other treatment for NSCLC, or had any history of cancer or prior radiotherapy to the chest area were excluded from the study. In all enrolled patients, 14 cases could not tolerate surgery because of comorbidities such as cardiovascular disease, chronic obstructive pulmonary disease (COPD), and severe diabetes. A central tumor was defined as being within 2 cm of the proximal bronchial tree, heart, great vessels, trachea, or other mediastinal structures; 3 patients (10.3%) had what were regarded as central tumors. The cost was calculated in US dollars, the cost during treatment was defined as the total cost of examination before treatment, simulation, and body ɤ-SRS treatment and the cost thorough 5 years was defined as the total cost of follow-up evaluations, treatment for any progression and adverse effect from the completion of body ɤ-SRS treatment to the end of the 5th year (Table 1).

**Table 1:** Clinical Characteristics and Outcomes of the 29 Enrolled Patients.

**Characteristic**	**No. of Patients (%)**
**AGE, YEARS**	
Median(range)	71 (55–87)
**GENDER**	
Male	22 (75.9%)
Female	7 (24.1%)
**ECOG PERFORMANCE STATUS**	
0	6 (20.7%)
1	14 (48.3%)
2	9 (31.0%)
**SABR INDICATION**	
Not candidate for surgery	14 (48.3%)
Refuse surgery	15 (51.7%)
**STAGE**	
Ia	16 (55.2%)
Ib	11 (37.9%)
IIa	2 (6.9%)
**HISTOLOGY**	
Adenocarcinoma	7 (24.1%)
Squamous cell	8 (27.6%)
MDT	14 (48.3%)
**LOCATION**	
Central	3 (10.3%)
Peripheral	26 (89.7%)
**CUMULATIVE INITIAL EVENTS**	
LR	3 (10.3%)
RR	8 (27.6%)
DM	11 (37.9%)
**DEATH**	
Due to Lung cancer	12 (41.4%)
Due to other disease	3 (10.3%)
Unknown	1 (3.4%)
Median PFS (95% CI), month	57.0 (37.6–76.4)
Median OS (95% CI), month	88.0 (35.7–140.3)
Median Cost during treatment (range), dollar	4,838 (4,615–4,923)
Median Cost through 5 years (range), dollar	36,960(9,920–56,824)

*ECOG, Eastern Cooperative Oncology Group; SABR, Stereotactic Ablative Radiotherapy; LR, Local recurrence; RR, Regional recurrence; DM, Distant metastasis; PFS, Progression free survival time; OS, Overall survival time*.

We declared that this study was according to the principles of Helsinki Declaration; the current study was approved by the ethics committee at the Airforce General Hospital, and all patients who agreed to attend the research were required to sign the ethical approval.

### Body ɤ-SRS

The patients enrolled in the study were immobilized with a stereotactic body frame and a vacuum pillow to ensure reproducible body position during simulation and treatment, and the patients breathed naturally without any breath control. The simulation of CT scan included the whole lung tissue and covered the area from neck midline to 3 cm under the diaphragm, with 5 mm slice thickness, 5 mm slice gap and a 5 s interval in scanning (9, 10).

We generated all the target volumes in the lung window CT. The GTV was the original tumor volume and the CTV was generated with a 5-mm margin around the GTV in all directions. The PTV was generated with a 10-mm margin around the GTV in all directions. Low-speed CT was used, regardless of respiratory movement on inside target volume (ITV). Limited by technological factors, a respiratory gating system was not used in the body ɤ-SRS treatment.

The treatment planning software was named Unicorn 3-D (developed by OUR International Technology & Science Co., Ltd. Shenzhen, People's Republic of China). Seventy Gy/10fractions (F) at a 70% isodose curve is the primary description dose standard and must cover at least 80% of GTV, while 60Gy/10F and 50Gy/10F at 60 and 50% isodose curves must cover at least 90% of CTV and whole PTV, respectively. The body ɤ-SRS was performed once every day, 5 times in 1 week and completed in 2 weeks (11).

Before each body ɤ-SRS treatment, the patients were to be scanned by CT to perform position verification. After the images were sent to the planning system, we grafted the previously designed target to the images and then compared the dose distribution as well as the dose to the target volume coverage between the positioning image and the validating image. A DVH diagram was used to verify the coincidence degree of target dose coverage. If at least 100% of PTV, 90% of CTV and 80% of GTV could not be covered by 50, 60, and 70% isodose curves, respectively, re-planning and re-verification were necessary. The decisions to re-plan and re-verify were made by physicians and patients together. The patients validated the posture 2–3 times during the treatment to ensure the accuracy of the positioning, planning, and treatment process.

### Follow-up Evaluations

The primary end points are overall survival (OS), progression-free survival (PFS) and the cost during treatment along with the cost through 5 years. Patients underwent physical examination and a chest CT scan at the end of 1st, 3rd and 6th months after treatment in the first half year, every 6 months for the next 2 years, and then annually. PET/CT was also used in all patients between 3 and 6 months after the completion of the body ɤ-SRS treatment. Approximately 50% (16/29) of the patients underwent subsequent PET/CT to further confirm the recurrent disease. LR was defined as progressive soft tissue abnormalities with CT evidence in the same lobe and the SUVmax>5 on PET/CT images obtained >3 months after SRS. Biopsy was the best choice to confirm any recurrence or suspected disease. RECIST criteria were employed to assess the therapeutic effect. In our study, we defined the RR as any lymphatic failure in the chest but, limited by medical conditions, endobronchial ultrasound-guided trans-bronchial needle aspiration (EBUS-TBNA) was not used in staging. DM was defined as any recurrence outside the chest or any disease recurrence in a different lobe. Acute adverse effect was defined as occurring within 6 months after treatment and late adverse effect was defined as occurring later than 6 months. Common Terminology Criteria for Adverse Events (CTCAE) Version 3.0 (U.S department of health and human services, National Institutes of Health National Cancer Institute) was used to evaluate radiation-induced adverse effect.

### Statistical Analysis

OS analysis was calculated from the start date of body ɤ-SRS to the date of death or last follow-up. PFS including any disease recurrence (LR, RR, and DM) was calculated from the start date of body ɤ-SRS to the date of the first disease failure or death. The date of disease recurrence was the date of the first PET/CT or CT image that demonstrated abnormalities.

All statistical analyses were performed using SPSS statistical software (version 24.0; IBM Corporation, Armonk, NY). The median follow-up was computed using the reverse Kaplan-Meier method. The OS curve was determined using the Kaplan-Meier method. Univariate analysis and multivariate analysis were performed by the Cox proportional hazards model, and all significance tests were 2-tailed with a *P*-value < 0.05 considered to be statistically significant.

## Results

From 2007–2010, 29 consecutive patients were recorded in the prospective body ɤ-SRS database (10). Of these, 14 (48.3%) patients were not candidates for surgery and 15 (51.7%) patients refused surgery. Fifteen (51.7%) patients were confirmed by histological evidence, 14 (48.3%) patients were confirmed by MDT. In all patients, 12 (41.4%) patients died of lung cancer, 3 (10.3%) patients died of other diseases and 1 (3.4%) patient died of unknown reason (Table 1). Owing to further decreased statistical power from the small sample size, sub-analysis could not be reliably performed.

**Figure 1:**
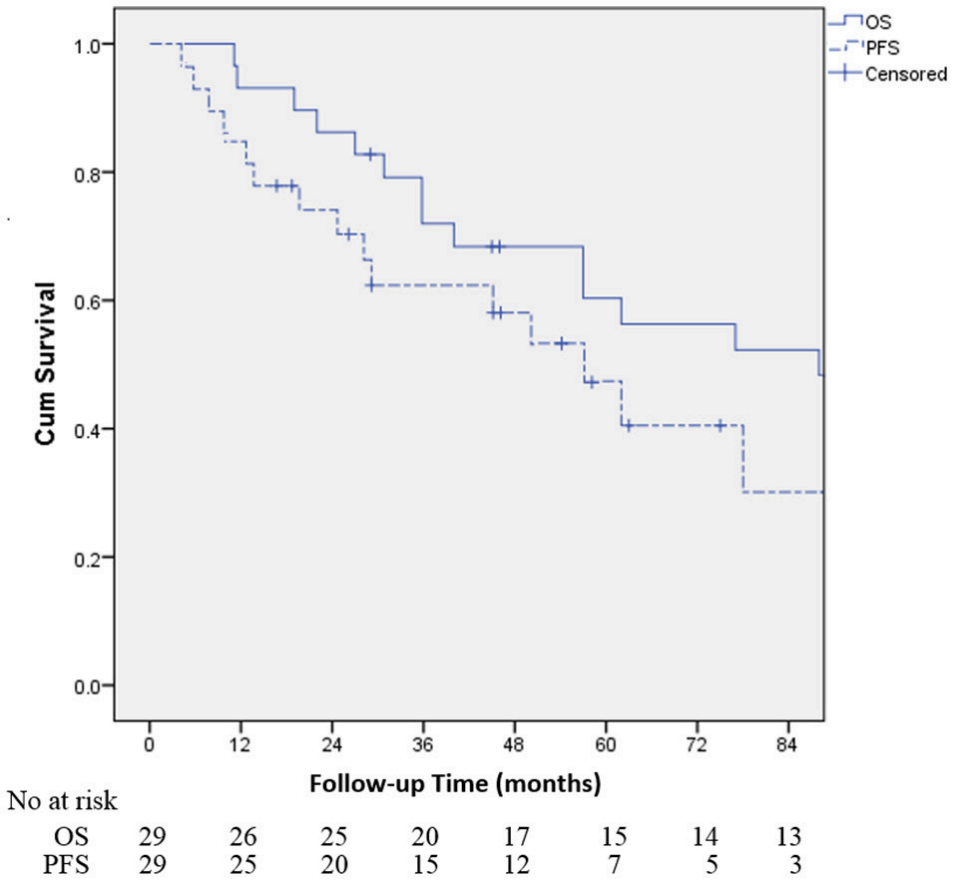
Kaplan-Meier curves for the cohort illustrating for overall survival (OS) and progression-free survival (PFS).

**Figure 2:**
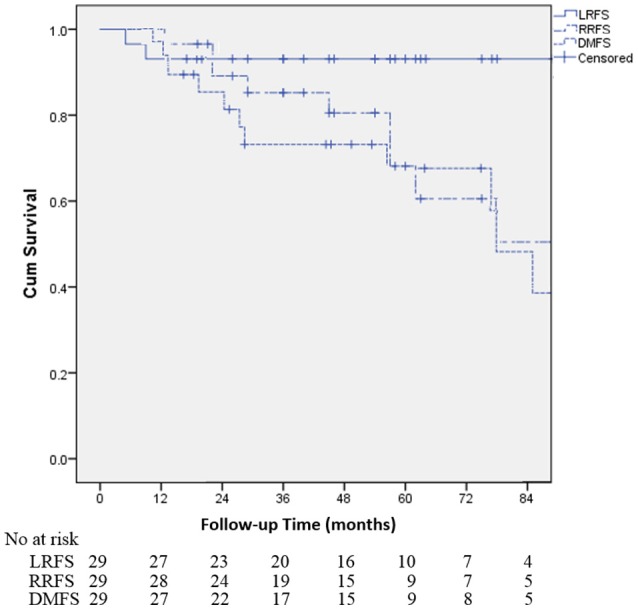
Kaplan-Meier curves for the cohort illustrating for local recurrence-free survival (LRFS), regional recurrence-free survival (RRFS) and distant metastases-free survival (DMFS).

Table 2 demonstrates that the estimated 1-, 3-, and 5-year OS rates were 93.1, 72.0, and 60.3%, respectively, and those for PFS rates were 86.2, 64.2 and 48.8%, respectively, (Figure 1). Estimated 5-year cumulative rates of LR, RR, and DM were 10.9, 21.4, and 29.0%, respectively, (Figure 2). The median follow-up time was 62.0 months (range, 11.1–140.3) and 22 (75.9%) patients had developed disease recurrence. The initial disease recurrence manifested as LR in 3 patients (10.3%), RR in 8 patients (27.6%) and DM in 11 patients (37.9%). Of the 3 patients who had LR as their first event, 2 (66.7%) patients had a very short time to LR (5.0 months and 9.0 months, respectively). Three (10.3%) patients with LR had synchronous RR and DM. One patient underwent subsequent body ɤ-SRS and chemotherapy and was still alive (123.6 months after the completion of initial body ɤ-SRS; the other 2 cases only received chemotherapy and died at 11.1 and 62.0 months after initial body ɤ-SRS). The most frequent initial sites of distant metastases were lung (36.4%) and bone (36.4%), while other sites included the brain, liver, adrenal gland and distant lymph nodes. The median cost of the body ɤ-SRS during treatment was 4,838 (range 4,615–4,923) dollars and median cost through 5 years was 36,960 (range 9,920–56,824) dollars (Table 1).

**Table 2:** Patterns of Failure and Survival after SABR.

**Event^**a**^**	**Actual Incidence%**	^****b****^**Estimated Cumulative Incidence%**		
		**1 year**	**3 years**	**5 years**
OS^b^		93.1	72.0	60.3
PFS^b^		86.2	64.2	48.8
Local disease recurrence	10.3	6.9	10.9	10.9
Regional disease recurrence	27.6	3.4	7.2	21.4
Distant metastases	37.9	3.6	22.6	29.0
Any progression	55.2	13.8	35.8	51.2

*95% CI, 95% Confidence interval; PFS: Progression free survival; OS, Overall survival; SABR, Stereotactic ablative radiotherapy*.

*^a^Any progression as the first event was calculated, and subsequent disease recurrence was not estimated*.

b *PFS, OS and Estimated cumulative incidence were calculated using the conventional Kaplan-Meier method*.

Table 3 demonstrates multivariate Cox proportional hazards modeling to examine factors associated with each of the OS, PFS, Local recurrence-free survival (LRFS), Regional recurrence-free survival (RRFS), and Distant metastasis-free survival (DMFS). Based on Cox proportional hazards modeling, ECOG, tumor size, histology and stage were associated with worse OS (*p* < 0.05), but no factors were associated with PFS, LRFS, RRFS or DMFS (*p* > 0.05).

**Table 3:** COX proportional hazards model results.

	**OS**				**PFS**				**LRFS**				**RRFS**				**DMFS**			
		**95%CI**				**95%CI**				**95%CI**				**95%CI**				**95%CI**		
	**HR**	**Lower**	**Upper**	***P***	**HR**	**Lower**	**Upper**	***P***	**HR**	**Lower**	**Upper**	***P***	**HR**	**Lower**	**Upper**	***P***	**HR**	**Lower**	**Upper**	***P***
Gender	2.0	0.4	11.6	0.420	0.6	0.2	2.6	0.527	4.1	0.0	6.8	0.980	0.1	0.0	315.2	0.589	1.8	0.3	13.6	0.547
Age	1.1	1.0	1.2	0.244	1.1	0.9	1.2	0.380	0.8	0.0	30.8	0.957	1.2	0.3	4.8	0.806	1.0	0.9	1.2	0.646
ECOG	1.0	1.0	1.2	*0.002*	1.4	0.0	5.6	0.582	1.0	0.0	2.3	0.991	1.0	0.0	158.1	0.349	1.0	0.0	1.5	0.074
Tumor Size	1.1	0.0	1.5	*0.008*	1.7	1.1	4.9	0.754	1.0	0.0	1.5	0.908	1.0	0.0	712.8	0.383	1.1	0.1	14.2	0.975
Histology	24.1	1.4	413.2	*0.004*	3.2	0.4	26.7	0.299	48.2	0.0	75.8	0.983	76.4	0.0	188.2	0.312	2.9	0.2	39.1	0.077
SABR indication	0.4	0.1	2.6	0.345	0.2	0.0	1.3	0.094	0.0	0.0	0.1	0.955	0.0	0.0	0.0	0.653	0.6	0.1	4.6	0.603
Location	19.4	0.0	319.6	0.354	0.0	0.0	0.0	0.964	73.5	0.0	156.1	0.917	18.8	0.0	52.2	0.413	0.0	0.0	0.0	0.973
Dose Fraction	450.8	0.0	678.6	0.985	0.0	0.0	0.0	0.963	12.4	0.0	56.8	0.939	79.8	0.0	131.1	0.441	0.0	0.0	0.0	0.973
GTV Volume	1.1	1.0	1.1	0.130	1.0	1.0	1.1	0.255	1.7	0.0	215.1	0.822	1.3	0.8	2.2	0.333	1.0	0.9	1.1	0.834
Stage	1.0	0.0	1.0	*0.004*	1.8	0.0	38.7	0.504	1.0	1.0	1.0	0.992	1.0	0.0	1.0	0.174	1.1	0.0	71.6	0.125

*ECOG, Eastern Cooperative Oncology Group; SABR, Stereotactic Ablative Radiotherapy; GTV, Gross target volume; OS, Overall survival time; PFS, Progression free survival time; LRFS, Local recurrence free survival time; RRFS, Regional recurrence free survival time; DMFS, Distant metastasis free survival time; CI, Confidence interval*.

Table 4 demonstrates that the most frequent side effects were radiation pneumonia, fatigue and dermatitis. Toxicity was classified according to the CTCAE 3.0. In all, only 2 of the 29 patients (6.9%) experienced grade 3 treatment-related adverse events (2 patients with radiation pneumonia). About half of patients had radiation-induced asymptomatic imaging changes during follow-up; grade 1 radiation pneumonitis (51.7%) was the most frequent (including grade 1 pulmonary fibrosis). No patient experienced grade 4 or 5 toxicity.

**Table 4:** Adverse Effects after SABR^a^.

**Adverse Effect**	**Grade 1 No. (%)**	**Grade 2 No. (%)**	**Grade 3 No. (%)**
^**b**^**ACUTE ADVERSE EVENT**			
Dermatitis	2 (6.9%)	0	0
Fatigue	3 (10.3%)	2 (6.9%)	0
Pneumonia	6 (20.7%)	0	2 (6.9%)
Nausea	0	1 (3.5%)	0
Anemia	0	1 (3.5%)	0
Total	11 (37.9%)	4 (13.8%)	2 (6.9%)
^b^**LATE ADVERSE EVENT**			
Fatigue	4 (13.8%)	2 (6.9%)	0
Pulmonary fibrosis	9 (31.0%)	0	0
Bone	1 (3.5%)	1 (3.5%)	0
Total	14 (48.3%)	3 (10.3%)	0

*SABR, Stereotactic ablative radiotherapy*.

a *Toxicity was graded according to the National Cancer Institute Common Terminology Criteria for Adverse Events (Version 3.0)*.

*^b^ Each symptom was scored separately. An acute event was defined as occurring within 6 months after SABR; late event was defined as one occurring >6 months after SABR*.

Twenty-eight patients (96.6%) accepted CT offline verification before the 1st and 6th treatment, respectively, two times in all. Only 1 patient (3.4%) accepted verification three times, because of re-planning (the first time is before the 1st treatment, the others are before 6th treatment).

## Discussion

To our knowledge, this report is the first analysis of data with long-term follow-up for the body γ-SRS in early stage NSCLC, and also the first report to evaluate the cost-effectiveness of the body γ-SRS. However, because of the small patient sample size, the findings must be interpreted cautiously. Our study demonstrated that body γ-SRS can achieve 90.1% of 5-year LC, 60.3% of 5-year OS and 48.8% 5-year PFS in early stage NSCLC with tolerable toxicity. The OS and LRFS are especially promising. These data are comparable with other advanced SRS technologies and surgical resection (12–15).

The optimal dose regimen for the body γ-SRS is still unknown. Corso et al. (16) analyzed the dose prescriptions and trends of SRS for 5246 patients with stage I NSCLC from 2004 to 2011 in the United States. Ninety-four percent of patients had a biologically effective dose (BED10) ≥100 Gy applied. The most common prescriptions were 54Gy/3F, 60Gy/3F, 48Gy/4F, and 50Gy/5F. The utilization of 54-60 Gy/3F decreased from 47.9% in 2006 to 27.9% in 2011. On the contrary, the utilization of 50Gy/5F increased from 3.1% in 2006 to 20.4% in 2011. The possible reason for these trends may be the concern for increased adverse effect with higher BED. In our study, with regimens of 70Gy/10F for GTV and 50Gy/10F for PTV, we achieved excellent LC as reported in another similar study (17) with limited toxicity, but the RR and DM were higher. We did not find associated factors with RRFS and DMFS, but the most probable concerns are mediastinal lymph node stage and BED for target volume. Limited by medical conditions, we did not use the EBUS in staging, but the EBUS (18–20) is quite valuable in the diagnosis of mediastinal lymph nodes. Furthermore, the nodal stage and treatment are associated with the RR (21). On the other side, Zhao reported that a higher radiation dose delivered to the PTV predicts for better local/lobar control (22). To our knowledge, it is still unclear whether there is an association between the radiation dosage for PTV and RR or DM. Lee reported that no patients experienced LR with tumors < 2 cm, without consideration of dose in SABR for medically inoperable stage I NSCLC. For tumors in which there was a diameter > 2cm, the escalated BED was associated with a higher LC rate (23). Given the difference in fractionation of SABR, our team is focusing our fraction size between 7 and 10 Gy to GTV for patients with tumor diameters ≤ 2cm following body γ-SRS.

In our study, no patients experienced grade 4 or 5 toxicity and only 2 (6.9%) cases experienced grade 3 radiation-induced pneumonia. The author thinks this is due to the special dose distribution of body ɤ-SRS, which can generate a conformal isodose distribution like radioactive seed implantation with delivery of the highest dose to the GTV and only a minimized dose to surrounding organs at risk. van Baardwijk et al. (24) reported lower to a certain extent, but more uniform doses distribution to the whole PTV may be sufficient to get a similar LC rate, and was potentially beneficial to central malignant tumors in the vicinity of critical structures. A phase II randomized clinical trial comparing body ɤ-SRS vs. helical tomotherapy-based SABR for early stage or isolated recurrent lung parenchymal NSCLC is ongoing in our institution.

The median cost of body ɤ-SRS during treatment was 4,838 (range 4,615–4,923) dollars, the median cost through 5 years was 36,960 (range 9,920–56,824) dollars, and the estimated 5-year OS and LC rates were 60.3 and 90.1%, respectively. It was less expensive and generated a similar outcome compared with other SABR technologies and surgery. Smith et al. (25) analyzed cost data from the Surveillance, Epidemiology, and End Results (SEER)-Medicare population, and found the mean weighted costs throughout 5 years for SABR and sublobar resection was 55,120 dollars and 77,964 dollars (*p* < 0.001); the costs for SABR were lower than those for sublobar resection, both for the pre-treatment phase (7838 vs. 9615 dollars, *P* = 0.02) and the treatment phase (12,436 vs. 26,522 dollars, *P* < 0.001). Lanni et al. (26) reported 86 patients with stage I NSCLC were treated with either SABR (*n* = 45) or external beam radiation therapy (EBRT) (*n* = 41); SABR was significantly less expensive (10,616 vs. 13,639 dollars, *P* < 0.01). Shah et al. (27) reported that the mean cost for SABR is 42,094 dollars. Whether considering cost through 5 years or cost during the treatment phase, the body ɤ-SRS is less costly and similarly effective with other SABR technologies and surgery, which is attractive especially in developing nations and underdeveloped countries.

Some limitations of body ɤ-SRS and our study also should be improved. In our study, the limited number of patients is the main defect, and we did not use the 4-dimensional CT or any motion management during simulation and treatment. We individually reviewed 3 patients who had initial LR. All tumors located at the right lower lobe had more significant breath movement. And the local recurrences all occurred within 1 cm of the PTV, where the BED10 is around 75Gy. The 5 mm margin between GTV and CTV, or 10 mm margin between GTV and PTV may not be enough for these lesions. Yang et al. (28) reported that more complicated and stricter measurements for the uncertainties and margins of SABR are warranted. Our clinical data showed that body γ-SRS can accurately irradiate most of the time in the absence of 4-dimensional CT. But the intrafraction motion could potentially lead to hot and cold spots in the tumor and, as a result, the 4-dimensional CT, the motion management and the cone beam CT should be involved in the body ɤ-SRS to narrow the scope, improve the dose for the target volume and to ensure the quality of the treatment in the future. The EBUS-TBNA should also be considered in staging.

## Conclusions

With existing clinical data, the body ɤ-SRS is an effective treatment option for patients with medically inoperable early stage NSCLC or patients who prefer not to have an operation, as they may derive benefits from the minimized toxicity. Due to excellent cost effectiveness, the availability of body ɤ-SRS will expand, especially in developing nations and underdeveloped countries.

## Author Contributions

HL: formal analysis, investigation, resources, data curation, writing (original draft, review, and editing), and visualization. JL: formal analysis, resources and writing (original draft, review, and editing). XW: investigation, data curation, and writing (review and editing). HP: data checking, statistical analysis and reporting, and writing (review and editing). YD: investigation, data curation, and writing (review and editing). GR, PL, XC, XK, and CL: investigation, resources, writing (review and editing), and supervision. YW and TX: conceptualization, methodology, formal analysis, validation, investigation, data curation, writing (original draft, review and editing), visualization, supervision, project administration, and funding acquisition.

### Conflict of Interest Statement

The authors declare that the research was conducted in the absence of any commercial or financial relationships that could be construed as a potential conflict of interest.
